# Aquaporin 4 and the endocannabinoid system: a potential therapeutic target in brain injury

**DOI:** 10.1007/s00221-024-06896-7

**Published:** 2024-07-23

**Authors:** Ari Misael Martínez-Torres, Julio Morán

**Affiliations:** División de Neurociencias, Instituto de Fisiología Celular, Universidad Nacional Autónoma de México, Coyoacán, Apartado Postal 70-253, 04510 Ciudad de Mexico, México

**Keywords:** Cannabinoids, Neuroprotection, Edema, AQP4, Brain injury

## Abstract

Brain edema is a critical complication arising from stroke and traumatic brain injury (TBI) with an important impact on patient recovery and can lead to long-term consequences. Therapeutic options to reduce edema progression are limited with variable patient outcomes. Aquaporin 4 (AQP4) is a water channel that allows bidirectional water diffusion across the astrocyte membrane and participates in the distinct phases of cerebral edema. The absence or inhibition of this channel has been demonstrated to ameliorate edema and brain damage. The endocannabinoid system (ECS) is a neuromodulator system with a wide expression in the brain and its activation has shown neuroprotective properties in diverse models of neuronal damage. This review describes and discusses the major features of ECS and AQP4 and their role during brain damage, observing that ECS stimulation reduces edema and injury size in diverse models of brain damage, however, the relationship between AQP4 expression and dynamics and ECS activation remains unclear. The research on these topics holds promising therapeutic implications for the treatment of brain edema following stroke and TBI.

## Introduction

In events such as stroke or traumatic brain injury (TBI), patient care time is essential to limit brain damage and improve survival. The magnitude of damage in both pathologies is determined by oxidative stress, the pro-inflammatory state, and the formation of cerebral edema. This last event is a decisive factor in the expansion of the lesion, defining the prognosis in both pathologies. Edema is the accumulation of fluid in the intracellular and interstitial space, resulting from a series of changes in cellular homeostasis that promote the evolution of ischemia and TBI (Jha et al. 2019).

Under physiological conditions, the fluid in the brain is distributed across four compartments; cerebrospinal fluid (CSF) compromising 80 to 100 ml, blood accounting for another 80 to 100 ml, intracellular fluid containing 100–130 ml and interstitial fluid making up 120–150 ml. Anatomically, the brain is contained in a rigid cranial vault, which does not allow the increase or decrease of its volume, implying that any change in the size of any of the compartments will generate changes in the rest of the compartments, affecting the intracranial pressure and brain structures (Stokum et al. 2016). The progressive increase in intracranial pressure can aggravate the initial damage, causing brain herniations and eventually the death of the patient.

## Blood–brain barrier and edema

The blood–brain barrier (BBB) is a specialized microvascular structure that forms brain capillaries, composed of endothelial cells, pericytes, and astrocytes. Under physiological conditions, BBB represents a physical and metabolic filter between blood glial cells and the extracellular fluid circulating in the brain. The BBB prevents the entry of pathogens (viruses, bacteria, parasites), toxins, and solutes, and controls the circulating volume in the brain (Sweeney et al. 2019). However, under acute damage conditions, BBB rupture determines the formation and evolution of edema, lesion expansion, and death (Chu et al. 2016; Dunn et al. 2021).

After an acute brain injury, three distinct phases of edema have been detected. Anoxic edema occurs minutes after injury and is characterized by water influx into astrocytes and neuronal dendrites. This event is due to a failure of ionic gradients secondary to decreased energy (ATP) production in the cell. Subsequently, a second phase occurs, the ionic edema, which is caused by the failure in the ionic gradients of the endothelial cells that make up the BBB, which determines the damage to the tight junctions that compose it, increasing the entry of water into the intracellular and interstitial space. In this phase, neuronal death begins to be observed (Jha et al. 2021; Han et al. 2023). Once the BBB has been completely uncoupled, vasogenic edema occurs. This type of edema is characterized by the entry of proteins into the brain tissue that under physiological conditions normally does not occur, as is the case for albumin. This condition generates a large movement of water, which increases the hypertonicity of astrocytes leading to neuronal death and expanding the lesion to areas that initially were not damaged (Stokum et al. 2016). The movement of water takes place through a group of molecules located in the plasma membrane known as aquaporins (AQP), among which aquaporin 4 plays a critical role.

## Aquaporins in brain

The study of edema formation during acute damage events in brain tissue led to the discovery of a family of molecules with a fundamental role in the control of cell volume, the aquaporins (AQP). These are transmembrane channels that selectively allow the passive and bidirectional diffusion of water across the plasma membrane. The water diffusion is controlled by the osmotic gradient of the cell generated by ion channels and transporters (Clément et al. 2020).

Structurally, AQP weighs an average of 30 kDa and is composed of six transmembrane alpha-helix domains, five connecting loops, and a water-selective pore, which contains a consensus Asn-Pro-Ala (NPA) motif, responsible for the selectivity to the water molecule, and grouped in tetramers in the membrane to form the water channel. (Gonen and Walz 2006). Currently, thirteen aquaporins are known in mammals, four of them have been reported to be present in the central nervous system: AQP1, AQP4, AQP9 and, more recently, AQP11.

AQP1 is present in epithelial cells of the choroid plexus and participates in the formation of cerebrospinal fluid (CSF) in conjunction with AQP4. The lack of both aquaporins in knock-out animals reduces CSF production and significantly decreases CSF efflux and distensibility of the cerebral ventricular system, indicating their participation in CSF evacuation (Trillo-Contreras et al. 2019). In the hydrocephalus, the CSF accumulation in the cerebral ventricles and subarachnoid space has been observed a decrease in AQP1 mRNA levels and a redistribution of this protein in the choroidal epithelium, as a compensatory mechanism to reduce CSF production. In AQP1 KO animals, a lower ventricular dilation corroborated their participation in CSF homeostasis (Owler et al. 2010; Wang et al. 2011).

AQP9 is expressed in the membrane of astrocytes, ependymocytes, endothelial cells of the meninges, and dopaminergic neurons of the ventral tegmental area and substantia nigra, and recently it was reported their presence in microglial cells (Badaut et al. 2004; Zahl et al. 2023). This aquaporin shows lower levels than AQP4, but a similar pattern of expression. The function of this aquaporin in the brain remains unresolved though in peripheral organs this aquaporin seems to be involved in proinflammatory responses (Tesse et al. 2021; Mohammad et al. 2022). Recently, it was found that AQP9 KO mice subjected to MPP + intracerebral injection showed reduced levels of pro-inflammatory molecules (Zahl et al. 2023).

AQP11 is in expressed in the cerebellum, hippocampus, cerebral cortex, and choroidal plexus epithelial cells (Koike et al. 2016). The function of this aquaporin remains unclear, it was reported that down-regulation of AQP11 contributes to the reduction of brain edema, BBB disruption, and neurological impairment after intracerebral hemorrhage (Xi et al. 2018). Nevertheless, in the 1321N1 astrocytes cell line, the upregulation of this aquaporin in the endoplasmic reticulum and plasmatic membranes was reported after LPS treatment. This was linked to a hydrogen peroxide (peroxiporin) function by exporting H_2_O_2_ from intracellular organelles into the cytoplasm to extracellular compartments and reducing oxidative stress and lipid peroxidation (Amro et al. 2024).

## Physiological and pathological roles of aquaporin 4 in the brain

**Fig. 1:**
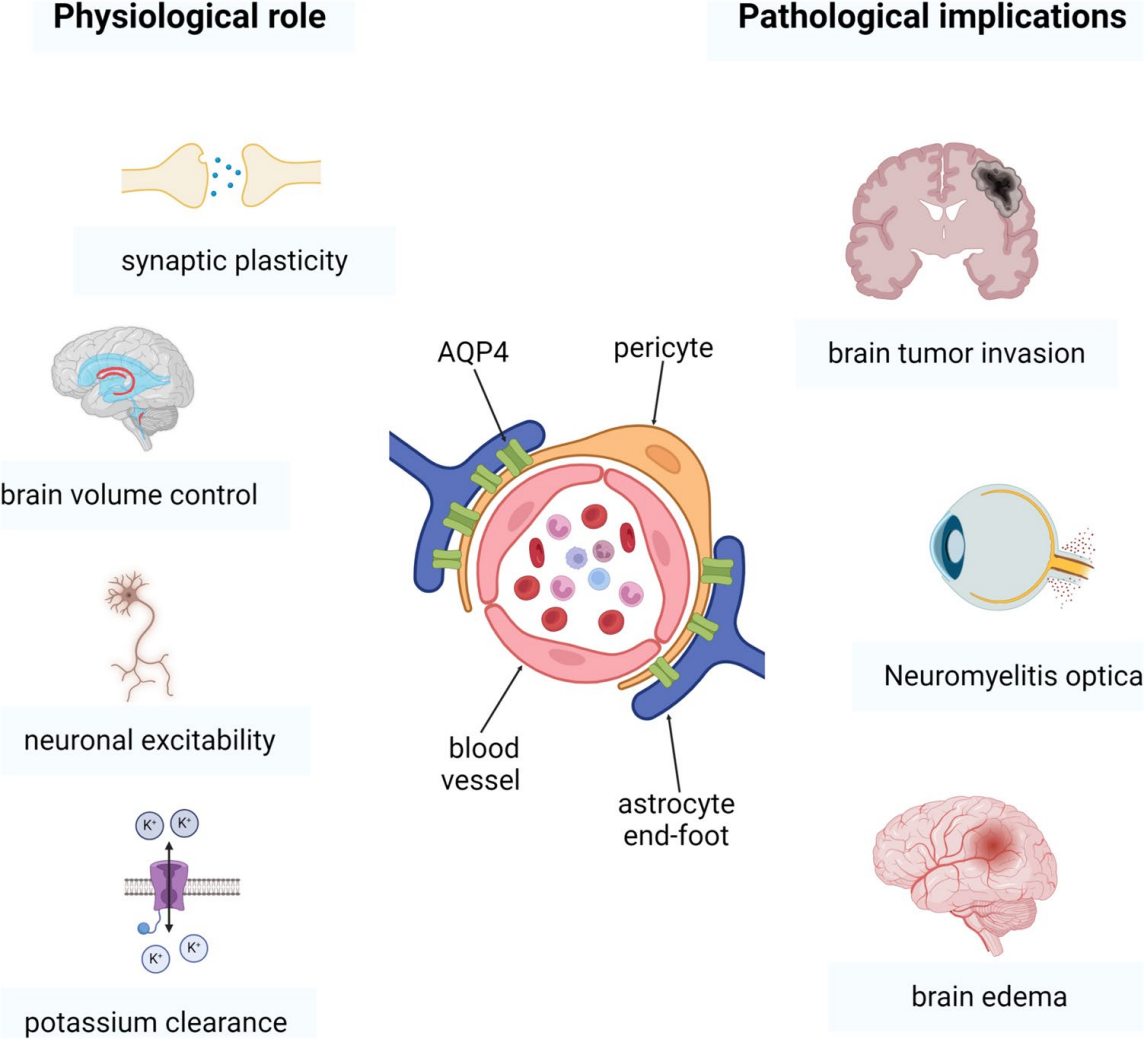
Physiological and pathological implications of AQP4 in the brain

Figure 1 describes the most important aspects of AQP4 in health and diseases in the brain. AQP4 participates in various physiological conditions in the brain, such as the control of extracellular volume. It was observed that mutant mice lacking AQP4 show a significant increase in extracellular volume and retention of water (Yao et al. 2008). Also, this aquaporin allows the water to flow through the blood vessels, allowing the clearance of interstitial fluid, interstitial wastes, and soluble proteins, forming part of a paravascular pathway for the clearance of interstitial fluids and wastes (lliff et al. 2012).

As mentioned above, AQP4 participates in the extracellular volume dynamics, highlighting its participation in the water efflux in astrocytes, acting in conjunction with the Na +  – K + /2Cl—cotransporter 1 (NKCC1), responsible for the astrocytic uptake of most of the potassium released by neurons after synaptic activation (Østby et al. 2009). In addition, during neuronal activation, AQP4 participates in the regulation of the extracellular space, decreasing the contraction of neuronal cells after synaptic activity and allowing water reflux (Haj-Yasein et al. 2012).

AQP4 has been identified as a potential regulator of synaptic plasticity. In AQP4 KO mice, a reduction in LTP was observed in CA1 and the perforant path-dentate gyrus (PP-DG) pathway. Delayed LTD was also observed in AQP4 KO mice (Skuas et al. 2011). In the postsynaptic membrane, the NMDA receptor (NMDAR) regulates LTP and LTD. NMDAR is activated by a change in extracellular pH. The cotransporter Na + /HCO3- regulates pH through water transport via the AQP4. In the absence of AQP4, the activity of the NMDAR is altered by variations in proton concentration and pH extracellular imbalances.

Astrocytes play a key role in potassium homeostasis and dysregulation in their levels can be related to LTP impairment. Studies have shown impaired potassium reuptake in mice lacking AQP4 and α-syntrophin. The increase in extracellular potassium leads to tonic depolarization and LTP impairment. The co-localization of AQP4 and Kir4.1 suggested an interaction between both proteins (Masaki et al. 2010). During the neuronal activity, the potassium released into the extracellular space compartment (ECS) is captured by Kir4.1 followed by the influx of water through AQP4 in the presynaptic space causing shrinkage of the ECS. The potassium taken by Kir4.1 is redistributed through the glial syncytium via gap junctions. AQP4 KO mice exhibited increased gap junctional coupling, leading to enhanced spatial buffering of potassium. The disruption of astrocyte gap junctional coupling impairs potassium homeostasis (Katoozi et al. 2017).

The lack of AQP4 also causes specific cognitive impairments. The AQP4 KO mice present a reduction in motivation and velocity to escape and a shorter swim path (Fan et al. 2013; Zhang et al. 2013). These animals exhibit a reduction in immobility in contextual fear conditioning (CFC) and indicate an impairment in associative fear memory formation (Li et al. 2012; Yang et al. 2013). The results obtained in the object placement (OP) test indicate that AQP4 KO mice present a deficit in object placement memory (Schafarman and Binder 2013).

In addition, in an animal model of epilepsy, the absence of the AQP4 anchoring protein worsens seizure severity (Amiry-Moghaddam et al. 2003). Therefore, the location of AQP4 in astrocyte end feet seems to play an important role in epileptogenesis, as changes in the localization of AQP4 or α-syntrophin or the absence of both seem to alter the onset and severity of seizures, but not the susceptibility to suffer crisis, reinforcing the idea of a probable role of AQP4 in neuronal excitability, a role not yet fully elucidated (Alvestad et al. 2013; Lee et al. 2012).

The AQP4 channel is implicated in CNS pathologies such as neuromyelitis optica spectrum disorder (DMOSD), a demyelinating and inflammatory disease detected in the spinal cord and the optical nerves. DNMO is caused by a pathogenic serum IgG antibody (NMO-IgG) against the AQP4 (Marignier et al. 2010). Diverse animal models have been developed in the study of this pathology, although administration of NMO-IgG leads to NMO-like disease (Remlinger et al. 2023). The antibody against AQP4 causes complement-dependent and complement-independent damage in astrocytes (Nishiyama et al. 2016). In the serum of AQP4-IgG positive DMOSD patients, monocytes showed increases in pro-inflammatory cytokines IL-6 and IL-1β and as non-classical monocytes (CD14^++^ and CD16^++^) were frequently observed in DMOSD serum patients (Kong et al. 2017). Leucocytes increase transmigration through the BBB and attack AQP4 in astrocytes in response to an increment in IL-6 levels (Chihara et al. 2011). One of the available treatments in this pathology is the monoclonal antibody against IL-6, Satralizumab, that reduces BBB dysfunction and activation of autoimmune T- and B-cells, and prevents neuroinflammation (Collongues et al. 2019).

In brain tumors such as astrocytoma, glioblastoma, and meningioma, the AQP4 expression is upregulated and participates in the pathogenesis of peritumoral edema and the increases of intracranial pressure. Also, AQP4 expression is related to tumor cell survival, migration, and invasion (Benham et al. 2022). Peritumoral edema increases neurological deficit and is a well-known negative prognosis factor. In intracranial meningiomas, AQP4 is overexpressed in conjunction with TRPV4 and participates in vasogenic edema (Faropoulos et al. 2021). Similar results were reported in glioblastoma multiforme, the increases in AQP4 expression corroborated with the peritumoral edema evaluated by magnetic resonance imaging (MRI) (Valente et al. 2022). In glioblastoma, siRNA-mediated down-regulation of AQP4 induced cell apoptosis, suggesting the role of AQP4 in tumor viability (Ding et al. 2013).

## Aquaporin 4 and cerebral edema

After brain injury, changes in the ionic balance determine the astrocyte swelling and the development of the distinct phases of edema. The depletion of ATP production after an ischemic event leads to Na^+^/K^+^-ATPase failure, sodium intracellular accumulation, and water influx through AQP4. In the same way, the metabotropic glutamate receptor induces astrocyte swelling by increasing Na + and K + influx (Illarionova et al. 2010). Specifically, mGluR5 showed an increase in co-expression with AQP4 in astrocytes in the penumbra after ischemia (Shi et al. 2017). Knockout of mGluR5 reduces BBB permeability and attenuates neurological dysfunction in TBI (Yang et al. 2017).

After ATP depletion, anaerobic metabolism produces extracellular lactate accumulation that reduces intracellular pH and ion fluxes and increases AQP4 expression in astrocytes (Morishima et al. 2008). Intracellular acidosis participates in cell swelling (Staub et al. 1990). Two types of transporters increase the activity after intracellular acidosis and participate in cellular swelling. The Na + /HCO3- transporter family (NBC) is the principal bicarbonate-dependent pH regulator in cultured astrocytes (Bavensse et al. 1997). The NBC current increases, after cerebral ischemia and treatment with specific NBC inhibitor S0859 reduces neuronal injury, astrocyte accumulation, and spatial memory impairment (Jia et al. 2023). The other group of membrane transport, the Na + /H + exchanger (NHE), particularly NHE1, facilitates H + efflux, accompanied by Na + influx, during brain injury and improves astrocyte swelling. The inhibition of absences of this protein reduces edema formation during ischemia (Kitayama et al. 2001; Yang et al. 2019).

**Fig. 2:**
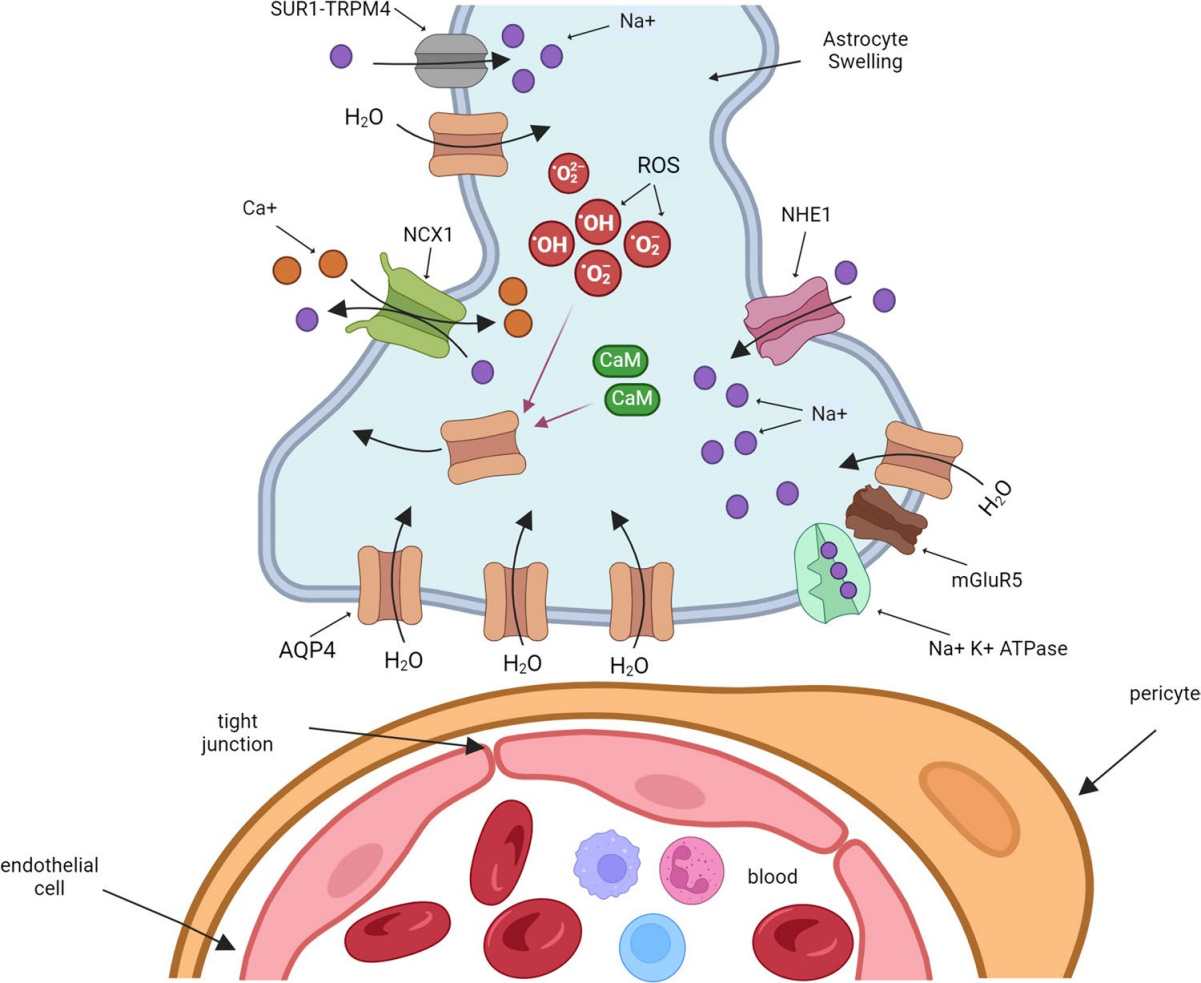
Molecular mechanism of edema formation in astrocytes endfeet. During the brain injury, the depletion of ATP leads to Na^+^/K^+^-ATPase failure and sodium intracellular accumulation. The glutamate receptor mGluR5 increases the Na^+^ and K^+^ influx. Intracellular acidosis increases the activity of the Na^+^/HCO3^−^ transporter family (NBC) and the Na^+^/H^+^ exchanger NHE1. SUR1-TRPM4 and the NCX1 Na^+^/Ca^+^ stimulate Ca^+^ influx. Increases in intracellular Ca^+^ and oxidative stress stimulate AQP4 translocation and facilitate water influx and astrocyte swelling

In addition, during ischemia, an increase in levels of SUR1-TRPM4 (permeable to divalent cations) and NCX1 (Na + /Ca + exchanger) was reported in astrocytes. The Na + influx through SUR1-TRPM4 promotes an increase in Ca + influx. This stimulates AQP4 translocation through calmodulin (CaM) activation and water influx (Ishida et al. 2022). The pharmacologic inhibition or the specific deletion of SUR1-TRPM4 or NCX1 reduces cerebral edema and neurological dysfunction (Stokum et al. 2023) (Fig. 2).

The AQP4 up-regulation during brain injury is controlled for oxidative stress. ROS production regulates AQP4 expression through the stimulation of various transcription factors. Increases in ROS levels activate genes containing the antioxidant response element (ARE) via NFE2-related factor-2 (NRF2). The promoter region of AQP4 contains putative ARE and their activation up-regulates AQP4 expression after brain injury (Mao et al. 2011; Zhao et al. 2005). The formation of the activating protein-1 (AP-1) in responses to oxidative stress leads to the activation of the p38 MAPK pathway and induces transcriptional up-regulation of AQP4 (Pan et al. 2010). Another transcription factor, nuclear factor κB (NF-κB) is up-regulated after an increase in ROS levels and induces the expression of AQP4 in astrocytes (Ito et al. 2006). Recently, in primary astrocyte cultures, the treatment with TNF-α increased AQP4 protein and mRNA levels, and cell volume by the stimulation of the NF-κB molecular pathway, Treatment with BAY11-7082, a specific inhibitor of the NF-κB pathway, reduced AQP4 levels, substantiating the participation of this transcription factor in AQP4 dynamic (Lu et al. 2022).

Other transcription factors have been pointed out in the transcriptional regulation of AQP4 during physiological and pathological conditions. Xiong and cols (2021, 2022) demonstrated the role of the HIF-1α factor in AQP4 activity under conditions of brain damage. Related results were found for other factors, such as NF-κB and Foxo3a under damage conditions (Sun et al. 2017; Kapoor et al. 2013; Zhang et al. 2019).

Treatment of primary astrocyte cultures with H2O2 induced an increase in membrane levels of AQP4. This event was independent of protein synthesis. The increase in membrane AQP4 levels was related to a rise in phosphorylation of Caveolin 1 (Cav-1), a protein involved in the subcellular distribution of AQP4, indicating that oxidative stress could indirectly regulate AQP4 activity (Bi et al. 2017). The relationship between oxidative stress and the activity of AQP4 was also observed in a brain ischemia model, where the treatment with antioxidants such as resveratrol and Edaravone markedly reduced AQP4 protein levels, limiting edema formation and lesion area (Alquisiras et al. 2020; Li et al. 2015).

AQP4 overexpression is observed during the first 24 h after experimental cerebral ischemia and, an event related to the increase in water brain content (Xiong et al. 2021; Dunn et al. 2021). The role of AQP4 in the initial stages of edema has revealed a significant increase in its expression within hours of injury onset, This elevated expression persists for at least 24 h, coinciding with the development of vasogenic edema (Katada et al. 2012; Zhang et al. 2015).

The involvement of AQP4 in acute brain injury has been validated using transgenic AQP4 knockout mice in a brain ischemia model. These studies demonstrated a decrease in edema formation and lesion area in KO AQP4 animals, demonstrating its involvement in edema formation during the first 24 h after the onset of damage (Sucha et al. 2022). Similar findings have been observed using TBI models, where the use of AQP4 KO rodents shows limited neuronal damage and a reduction in neurological deficit as compared to the control group (Liu et al. 2021). Also, the use of siRNAs for AQP4 has evidenced anti-edematous and neuroprotective effects in brain injury models (Wang et al. 2021; Guan et al. 2020; Zhang et al. 2019). Pharmacological studies have also been directed to evaluate the role of AQP4. In this regard, TGN-020, an AQP4 blocker, significantly reduces brain damage, edema development, and morphological changes in astrocytes (Cui et al. 2020; Sun et al. 2022; Li et al. 2024). Interestingly, the use of specific antibodies against AQP4 seems to have a dual effect. Administration of AQP4 antibody 30 min prior to inducing middle cerebral artery occlusion (MCAO) resulted in a significant increase in injury size and edema at 24 h post-occlusion compared to the control group. In contrast, AQP4 antibody 30 min after controlled cortical injury (CCI) reduced edema and hippocampal neuron loss at 24 h and 21 days post-injury. These contradictory results are due to the brain injury models used, the number of days post-injury before evaluation (21 days versus 1 day, respectively), and the unspecific neurotoxic action of the AQP4-IgG impairing the glutamate transport by down-regulating the glutamate transporter EEAT2, increases excitotoxicity and aggravates damage. (Juenemann et al. 2015; Xiong et al. 2022).

The neuroprotective activity of several molecules with antioxidant and anti-inflammatory properties in animal models of brain ischemia and TBI are associated with a reduction in AQP4 expression and activity. Natural antioxidants such as resveratrol have been shown to decrease the expression of this protein, and the formation of edema in animals, and improve neurological deficit (Alquisiras et al. 2020). The downregulation in AQP4 expression and decrease in edema formation have also been observed with Edaravone, a synthetic antioxidant that is clinically used in the treatment of brain stroke in Japan (Ren et al. 2019; Kikuchi et al. 2009).

## Endocannabinoid system and neuroprotection

The study of new therapeutic targets for the treatment of acute pathologies in the central nervous system has focused on studying neuromodulator systems with a wide presence in the brain that undergo changes in their expression and activity in brain diseases. Such is the case with the endocannabinoid system (ECS). The ECS comprises cannabinoid receptors type 1 and 2 (CB1 and CB2) and the endogenous ligands, the arachidonoyl ethanol amide (anandamide) and 2-arachidonoyl glycerol (2-AG), as well as enzymes responsible for their synthesis and degradation. The ECS has been related to neurodevelopment and plasticity, but they have also been linked to the protection of acute brain injury and neurodegenerative pathologies.

In contrast to CB2 receptors, the density and distribution of CB1 receptors are abundant in the brain, particularly in the cerebral cortex, basal ganglia, hippocampus, and cerebellum. They are in the presynaptic and postsynaptic terminals (Mackie 2005). On the other hand, CB2 receptors are expressed in microglia and vascular cells (Ramirez et al. 2012); however, it has been reported that under pro-inflammatory conditions, CB2 is also expressed in neurons where they can induce neuroprotective actions (Viscomi et al. 2009).

Cannabinoid receptors are 7-domain membrane G protein-coupled receptors (GPCRs) located in the cytoplasmic membrane. They modulate adenylate cyclase (AC) activity and cAMP levels through the activation of the stimulatory (Gαs) or inhibitory (Gαi) subunits, increasing the activity of AP-1 (activator protein 1) and some protein kinases, such as PKA that mediates the phosphorylation and activation of the ERK 1/2 signaling cascade (Bosier et al. 2008; Dalton and Howlett 2012). This signaling pathway can also be activated by CB1 internalization and recruitment of β-arrestin 1 and 2 (Ibsen et al. 2019; Grafinger et al. 2021).

The PI3K/Akt pathway seems to play a key role in the neuroprotective properties of the ECS (Tadijan, et al. 2022). Upon activation by endocannabinoids, the CB1 receptor triggers a cascade of events that activate Akt, a protein kinase with numerous downstream targets involved in cell survival, growth, and proliferation. This activation leads to the inhibition of pro-apoptotic factors and the promotion of neurotrophic factors, effectively protecting neurons from damage and promoting their survival (Massi, et al. 2010) (Ligresti et al. 2016; Hollville et al. 2019).

In addition to the PI3K/Akt pathway, peroxisome proliferator-activated receptors, particularly the PPAR-α subtype, contribute significantly to the neuroprotective effects of cannabinoids (Lago-Fernandez et al. 2021). PPARs are ligand-activated transcription factors that regulate gene expression, influencing various cellular processes, including lipid metabolism, inflammation, and cell survival (Sanjay et al. 2021; Khosropoor et al. 2023). Cannabinoids, acting as ligands, activate PPAR-α, suppressing inflammatory responses and upregulating neuroprotective genes (Iannotti and Vitale 2021; Neher et al. 2012). This modulation of gene expression contributes to the overall neuroprotective effects observed with cannabinoid use.

The intricate interplay between the PI3K/Akt pathway and PPARs highlights the complexity of cannabinoid-mediated neuroprotection (Ibeas et al. 2015; Kumar et al. 2022). While the PI3K/Akt pathway directly influences cell survival signaling, PPARs exert their effects by modulating gene expression, contributing to a neuroprotective environment (Bhunia et al. 2022). Understanding the specific mechanisms by which cannabinoids engage these pathways is crucial for developing targeted therapeutic strategies for neurodegenerative diseases.

The synthesis of endocannabinoids occurs through different pathways. In the case of anandamide, the metabolic routes may vary in each brain region. The first pathway is based on the hydrolysis of N-acyl phosphatidylethanolamines (NAPE) by a phospholipase D (PLD) (Schmid et al. 1983). The second route is the cleavage of the phosphodiester bond of NAPE by phospholipase C (PLC); subsequently, a dephosphorylation of phospho-anandamide produces anandamide (Liu et al. 2006). The synthesis of 2-AG starts with the activity of PLCβ, which hydrolyzes a PIP2 molecule, and the resulting diacylglycerol is hydrolyzed by Diacyl glycerol lipase (DAGL), finally generating 2-AG (Murataeva et al. 2014). There are other routes for the synthesis of endocannabinoids that are not yet fully elucidated.

The degradation of endocannabinoids is critical to maintain their endogenous balance. Anandamide can be metabolized by the fatty acid amino hydrolase (FAAH), which reduces a large amount of fatty acid amides (Cravvat et al., 1996). Another degradation pathway is through its oxidation by cyclooxygenase-2 (COX-2) which generates prostamides (Woodward et al. 2008). The degradation of 2-AG is mediated by the action of three enzymes: monoacylglycerol lipase (MAGL) and the alpha/beta domain hydrolases 6 and 12 (ABHD6 and 12). In addition, in the case of anandamide, 2-AG can also be oxidized by COX-2 (Blankman et al. 2007).

The neuroprotective activity of the ECS after stroke and TBI has been widely explored (Table 1). The phytocannabinoids cannabidiol (CBD) and THC (tetrahydro cannabidiol) were the first cannabinoids evaluated against brain injury. In a stroke model, the administration of CBD through the intracerebroventricular (i.c.v.) route at doses of 100 and 200 ng/kg 5 days before MCAO (middle cerebral artery occlusion), reduces infarction volume and the percentage of pyknotic neurons through the regulation of oxidant and inflammatory factors (Khaksar and Bigdeli 2017, Khaksar et al. 2022). Similarly, intraperitoneally administration of CBD at doses of 5 and 10 mg/kg after the MCAO model reduced infarction volume and neurological deficit (Meyer et al. 2022; Chen et al., 2024).

**Table 1:** Summary of reports about the ECS stimulation during brain injury and their effects on neurological impairment, brain swelling, BBB permeability, lesion or infarct volume, and AQP4 expression

Drug and dosage	Brain injury model	Effects reported	Reference
2-AG (2-arachidonoylglycerol) 5 mg/kg i.p	CHI (Close head injury)	↓ water content ↓lesion volume ↓neurological impairment ↓ hippocampal cell death	Panikashvili et al. 2001
2-AG 5 mg/kg i.p	CHI	↓ BBB permeability ↓ proinflammatory cytokines	Panikashvili et al., 2005
AM-404 0.015, 0.03, 0.1, 0.5, 1, 2 mg kg i.p THC (D9-Tetrahydrocannabinol) 0.015, 0.03, 0.1, 0.5, 1, 2 mg kg i.p	Transient global cerebral ischemia	↓ histological changes ↓ neurological impairment	Zani et al. 2007
O-1966 1,5 and 10 mg/kg i.p	MCAO (Middle Cerebral Artery Occlusion)	↓ BBB permeability ↓ infarct volume ↓ neurological impairment	Zhang et al. 2009
WIN-55,212–2 1 mg/kg i.p	MCAO	↓infarct volume	Hu et al. 2010
O-1966 5 mg/kg i.p	CCI (Controlled Cortical Impact)	↓neurological impairment ↓brain swelling ↓reactive microglia	Elliott et al. 2011
JWH-133 1.5 mg/kg i.p SR144528 3–5 mg/kg i.p	MCAO	↓neurological impairment ↓infarct volume ↓proinflammatory markers	Zarruk et al. 2012
WIN55,212–2 0.3 or 1 mg/kg i.p	MCAO	↓BBB disruption	Chi et al. 2012
0–1966 5 mg/kg i.p	CCI	↓ lesion volume ↓ neurological impairment ↓ neurodegeneration	Amenta et al. 2012
WIN-55,212–2 9 mg/kg i.v	MCAO	↓brain swelling ↓infarct volume	Sun et al. 2013
PF3845 2, 5 or 10 mg/kg i.p AM281 3 mg/kg i.p AM630 3 mg/kg i.p	CCI	↓ neurological impairment ↓ lesion volume ↑ AEA levels	Tchantchou et al. 2014
JZL184 16 mg/kg i.p URB591 0.3 mg/kg i.p	LFPI (Lateral Fluid Percussion Injury)	↓ neurological impairment ↓ BBB disruption ↓ proinflammatory cytokines	Katz et al. 2015
ACEA ((arachidonyl-2-chloroethylamide) 1 mg/kg i.p AM-251 1 mg/kg i.p	MCAO	↓ histological changes ↓infarct volume ↓ neurological impairment ↓ astroglial reactivity	Caltana et al. 2015
CBD (cannabidiol) 50,100 and 200 ng/rat i.c.v	MCAO	↓infarct volume	Khaksar and Bigdeli., 2017
JZL184 16 mg/kg i.p	LFPI	↓ neurological impairment ↓reactive astrocytes	Mayeux et al. 2017
JZL184 4 mg/kg i.p AM-251 3 mg/kg i.p	MCAO	↓infarct volume ↓brain swelling ↓neurological impairment	Rhamani et al.,2018
HU-910 0.1–10 mg/kg i.p HU-914 5 and 10 mg/kg i.p SR144528 1 mg/kg i.p AM630 1 mg/kg i.p	CHI	↓lesion volume ↓neurological impairment	Magid et al. 2019
ACEA 1.5 mg/kg i.p AM-215 1 mg/kg i.p	MCAO	↓infarct volume ↓neurological impairment ↓neuronal loss	Yang et al. 2020
MJN110 2.5 mg/kg i.p AM281 3 mg/kg i.p AM630 3 mg/kg i.p	CHI	↓ neurological impairment ↓ proinflammatory cytokines ↓ neuronal death ↑ 2-AG levels	Selvaraj et al. 2021
PF04457845 5 mg/kg i.p AM281 3 mg/kg I,p, AM630 3 mg/kg i.p	CHI	↓ neurological impairment ↓ proinflammatory cytokines ↓reactive astrocyte and microglia	Selvaraj et al. 2021
CBD 5,10,20, and 30 mg/kg i.p	CHI	↓ brain swelling ↓ BBB permeability ↓ neurological impairment ↓ AQP4 expression	Jiang et al. 2021
CBD (20 mg/ml) through a gelfoam sponge in the open contusion site CBD 40 mg/kg and 20 mg/kg i.p	CCI	↓neurological impairment ↓lesion volume ↓neuronal death ↓reactive microglia	Friedman et al. 2021
CBD 50,100 and 200 ng/rat i.c.v	MCAO	↓infarct volume ↓ histological changes ↑SOD and CAT activity ↓MDA levels	Khaksar et al. 2022
CBD 50,100 or 200 mg/kg orally	LFPI	↓neurological impairment ↓glutamate concentrations	Santiago-Castañeda et al. 2022
CDB 10 mg/kg i.p	MCAO	↓neurological impairment ↓neuronal degeneration ↓reactive microglia	Meyer et al. 2022
JWH133 1.5 mg/kg i.p SR144528 3.0 mg/kg i.p	CCI	↓lesion volume ↓neurological impairment ↑ myelinated axons and oligodendrocytes	Li et al. 2022
THC 3 mg/kg i.p	CCI	↓ neurological impairment	Song et al. 2022
VCE-004.8 10 and 20 mg/kg i.p	MCAO	↓infarct volume ↓neurological impairment ↓BBB permeability ↓proinflammatory cytokines	Lavayen et al. 2023
CBD 5 mg/kg i.p	MCAO	↓infarct volume ↓reactive microglía ↓proinflammatory cytokines	Chen et al.,2024
CBD 5 mg/kg i.p	LFPI	↓neurological impairment ↓AQP4 polarization	Dong et al. 2024

The administration of THC at doses of 1 mg/kg after injury reverted the behavioral alteration and the histological changes (Zani et al., 2009). In traumatic injury, oral treatment with CBD at doses of 50,100 and 200 mg/kg daily 7 days before injury improves sensorimotor functions and reduces excitotoxic (Santiago-Castañeda et al. 2022). The administration of CBD through a patch (20 mg/ml) was applied over the dura at the injury site and intraperitoneally at doses of 40 mg/kg after injury and 20 mg/kg the next 5 days. The combination of routes reduces the injury volume and increases the performance of the animals in the beam balance test and the mobility and recognition of novel objects (Friedman et al. 2021). Similar results in motor, memory, and cognitive functions were reported by Dong y cols. Furthermore, the treatment with THC 3 mg/kg i.p. for 3 days after traumatic brain injury in mice improved working memory and locomotor functions (Song et al. 2022).

The development of synthetic agonists and antagonists of the cannabinoid receptors contributed to the knowledge of the participation of ECS in brain injury. This clarified the contradictory results obtained in KO CB receptor animals during stroke, where deletion of either CB1 or CB2 receptors increased damage following experimental stroke, but the combined deletion of both receptors reduced infarct volume and improved neurological recovery in the same experimental model. The probable reason for this result is the activation of homeostatic pathways to compensate for the loss of both receptors (Ward et al. 2018; Parmentier-Batteur et al. 2002). The use of ACEA, a synthetic CB1 receptor agonist, in a model of ischemic stroke, reduced the infarct area and improved motor recovery, a phenomenon that was nullified with the co-administration of AM-251, an antagonist of CB1 (Caltana et al. 2015; Yang et al. 2020). After the TBI, treatment with the same agonist attenuated anxiety-like behavior (de la Tremblaye et al. 2021).

In addition, the administration of 2-AG, another synthetic cannabinoid, reduces the area of injury and neurological impairment and preserves BBB integrity in cerebral infarction (Panikashvili et al. 2006, 2001). In addition, the agonist of the CB1 receptor, WIN 55-212-2, administered orally or intraperitoneally in rats subjected to MCAO, reduced brain edema and infarct volume. These effects were mediated by the activity of the ERK 1/2 signaling pathway (Hu et al. 2010; Sun et al. 2013). Also, the intravenous administration of this agonist at the dose of 0.3 or 1.0 mg/kg attenuates BBB disruption after ischemia (Chi et al. 2012).

On the other hand, the CB2 receptor has been related to neuroinflammatory conditions. The involvement of this receptor in ischemic stroke and brain injury models has been demonstrated. For example, the use of O-1966, a CB2 receptor agonist, markedly reduced the neurological damage and the area of injury in a model of ischemia. Interestingly, this treatment also preserved the integrity of the BBB (Zhang et al. 2009). The use of 1.5 mg/kg of JWH-133 after MCAO reduces infarction area, microglial reactivity, and pro-inflammatory response (Zarruk et al. 2012). In TBI models, treatment with the same CB2 agonist at doses of 5 mg/kg at 2, 24, 48, and 72 h after injury reduced BBB disruption, neuronal degeneration, and improved performance in rotarod and open field test (Amenta et al. 2012). Elliot and cols. reported similar findings in motor and behavior tests, and a reduction in brain water content in the group treated with O-1966 at 48 h post-injury. Another CB2 agonist, VCE-004.8, exhibited similar effects at 72 h after ischemic stroke (Lavayen et al. 2023). Comparable results were obtained in animals subjected to traumatic brain injury and treated with HU-910, HU-914, and JWH133, selective CB2 receptor agonists (Magid et al. 2019; Li et al. 2022).

In addition, to the use of cannabinoid receptors agonists and antagonists, the inhibition of endocannabinoid degradation by using MJN110 and CPD-4645, inhibitors of the enzyme monoacylglycerol lipase, reduced neurological dysfunction and BBB permeability by increasing 2-AG levels during brain injury (Piro et al. 2018; Selvaraj et al. 2019). In permanent MCAO (PMCAO), the treatment with JZL-184 at a dose of 4 mg/kg reduces brain edema, infarction area, and motor dysfunction (Rahmani et al. 2018). Comparable results were reported with PF04457845, a fatty acid amide hydrolase inhibitor that increases anandamide (AEA) levels (Selvaraj et al. 2021). In rats subjected to TBI, the treatment with JZL184 (MAGL inhibitor) and URB597 (inhibitor of FAAH) improved neurological and behavioral impairment and reduced BBB disruption at 24 h after injury (Katz et al. 2015). A reduction in astrocyte activation and an increase in neurobehavioral recovery was reported with JZL184 at 14 days after TBI (Mayeux et al. 2017). Another FAAH inhibitor, PF3845, at doses of 2, 5, or 10 mg/kg i.p post-injury for 3 or 14 days, reduced lesion volume and impairments in motor function, working memory, and anxiety-like behavior (Tchantchou et al. 2014).

## ECS neuroprotection and AQP4

As previously mentioned, there is a solid line of research about the neuroprotective role of ECS in acute brain injury conditions (Table 1). Alternatively, AQP4 plays a fundamental role in edema formation and neurological damage. However, the relationship between AQP4 and CB1 and CB2 receptor activities has not been fully studied during brain injury. Lopez-Rodriguez et al. (2015b) described the involvement of CB receptors in minocycline-mediated protection against brain edema and neurological impairment using a model of TBI and AM-251 and AM-630, antagonists of the CB1 and CB2 receptors. Subsequently, this group reported the activation of CB1 and CB2 receptors and their relationship with edema during TBI, observing that CB1 receptor activation significantly decreased its expression at mRNA and protein levels during the first hours of the injury, while CB2 was overexpressed (Lopez-Rodriguez et al. 2015a). The observed changes in the expression of both receptors did not show a direct correlation with the formation of edema and levels of AQP4, which markedly increased 24 and 72 h after the injury and correlated with the increase in brain water content. This study is not conclusive because CB1 and CB2 receptors were not exogenously activated or blocked.

Recently, in a TBI model, it was reported that an increase in AQP4 expression was related to a reduction in the levels of the endocannabinoids 2-AG and AEA and an increase in their respective degradation enzymes MAGL and FAAH, indicating the importance of endocannabinoid metabolism during brain damage (Ahluwalia et al. 2023). In this regard, it was also shown that CBD administered before and after TBI reduced brain water content, prevented blood–brain barrier disruption, and decreased AQP4 expression and AQP4 positive cells (Jiang et al. 2021); however, this is a correlative study, and it is not clear whether CBD acted directly or indirectly to induce a reduction in AQP4 levels. Additionally, it is known that CBD has a low affinity for both cannabinoid receptors (CB1 and CB2) and its neuroprotective effects could be due to different mechanisms, including an increase of anandamide levels and the antioxidant properties of the molecule (Belardo et al. 2019). However, lately, it was reported that CBD treatment after traumatic brain injury facilitates the reduction of AQP4 polarization at 3 days post-TBI, improving glymphatic system functionality (Dong et al. 2024). These results suggest that the modulation of ECS may exert neuroprotective effects by regulating the AQP4 dynamic.

The participation of ECS in AQP4 expression has also been studied in human colon tissue, where the treatment with CBD and palmitoylethanolamide (PEA) reduces its expression under inflammatory conditions (Couch et al. 2019). These results were also obtained using oleoylethanolamine (OEA) in Caco-2 cells (Karwad et al. 2017). As mentioned above, despite this evidence, the role of ECS in AQP4 activity and dynamics during brain injury remains unclear.

## Antioxidant properties of ECS as a potential AQP4 activity regulator

The brain is particularly susceptible to oxidative stress due to its high oxygen consumption and limited antioxidant defense system. Oxidative stress is one of the main molecular events after acquired brain injury is caused by an imbalance between the formation of reactive oxygen species (ROS) and nitrogen (RNS) and their removal by the antioxidant systems, and leads to damage in proteins, DNA, and fatty acids (Merelli et al. 2021). The main ROS includes superoxide anion (O^−2^), hydroxyl (OH), singlet oxygen (O_2_), and hydrogen peroxide (H_2_O_2_), while RNS includes nitric oxide (NO) and peroxynitrite (ONOO). The main cellular sources of ROS/RNS are mitochondria, NADPH oxidase enzymes (NOX), nitric oxide synthase (NOS), cyclooxygenase (COX), and lipoxygenases (LOX), among others.

Some cannabinoids exhibit oxidant properties, using cyclic voltammetry assay, CBD, THC, HU’211, cannabinol, nabilone, and levanantrodol showed the capacity to donate or accept electrons under a variable voltage potential. This capacity was also observed for the antioxidant butylhydroxytoluene (BHT) (Hampson et al. 1998). Furthermore, in HT22 cells exposed to H_2_O_2,_ the treatment with anandamide (AEA) reduced the levels of intracellular ROS and oxidized glutathione (GSSG), NOX2 expression, and increased superoxide dismutase (SOD), and glutathione (GSH) levels, substantiating the ability to stimulate the antioxidant system (Jia et al. 2014).

The involvement of ECS in the regulation of oxidative stress in models of brain injury has been evaluated in several experimental models. Javed et al. (2016) investigated the neuroprotective potential of β-caryophyllene, a CB2 agonist, in a rotenone-induced Parkinson's disease animal model. Their findings indicated that CBP treatment effectively attenuated glutathione depletion and lipid peroxidation while enhancing the levels of antioxidant enzymes catalase and superoxide dismutase. These changes were associated with a significant reduction in neuronal loss within the substantia nigra, highlighting the potential therapeutic benefit of CBP in Parkinson's disease. In oligodendrocyte progenitor cells (OPCs) exposed to LPS/TNF-α, treatment with CBD attenuates ROS productions and apoptosis through a molecular mechanism that involves the endoplasmic reticulum (ER) stress (Mecha et al. 2012). Some other studies about oxidative stress modulation by ECS have been reported. For example, the use of the cannabinoid agonist WIN-55,212-2, in a quinolinic acid model that induces neurotoxicity in primary rat striatal cell cultures, prevented lipid peroxidation, ROS formation, and loss of cell viability (Rangel-Lopez et al. 2018). Additionally, an increase in anandamide level by URB591, a FAAH inhibitor, increased catalase and SOD activity and reverted the increases of ROS and MDA in BMEC cell cultures exposed to oxygen–glucose deprivation (OGD) (Wang et al. 2022). Also, in a model of chronic unpredictable stress (CUS) in rats, the URB597, another FAAH inhibitor, reduced lipid peroxidation and the observed depressive behavior (Tejeda-Martinez et al. 2021). Finally, in zebrafish subjected to acute restrain stress (ARS) the cannabinoid agonist, ACEA reduced the MDA levels and anxiety-like behaviors (Lucas Luz et al. 2021; Pinheiro et al.; 2022). Thus, as previously mentioned, the CB receptor stimulation or the inhibition of endocannabinoid degradation reduces oxidative stress through not fully understood intracellular pathways. In addition, the oxidative conditions regulate AQP4 expression and activity. It is therefore possible that the ECS could regulate AQP4 activity during brain injury through a regulation of the ROS/RNS production.

## Conclusions and future directions

**Fig. 3:**
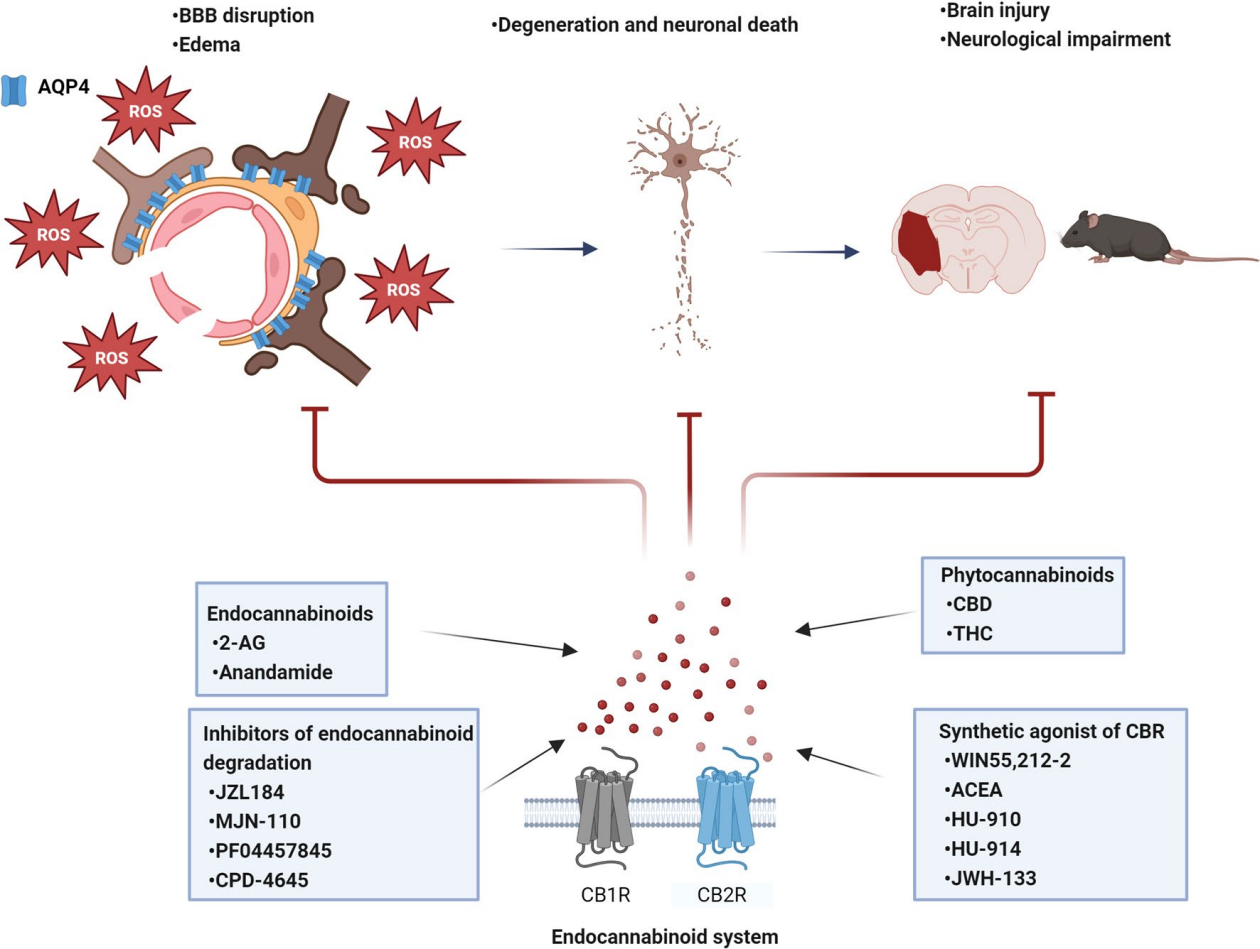
After the brain injury, the stimulation of the endocannabinoid system exerts a neuroprotective effect and attenuates edema and brain blood barrier (BBB) disruption through the reduction of oxidative stress and AQP4 activity. *2-AG* 2-arachidonoylglycerol; *CBD* cannabidiol; *THC* tetrahydrocannabidiol

As described before, previous works indicate that after brain injury, the stimulation of the ECS can reduce oxidative stress and limit BBB disruption, brain edema, and neuronal death, reducing injury extension and neurological dysfunction in animal models (Fig. 3). Aquaporin-4, a membrane protein localized in the terminal ends of astrocytes, plays a critical role in the regulation of cell volume and the integrity of the blood–brain barrier. AQP4 is overexpressed during the establishment of cerebral edema in acute damage events, such as brain trauma and cerebral ischemia. The molecular mechanism associated with AQP4 expression and its mobilization to the cell membrane is not fully understood; however, the involvement of oxidative stress is a critical event. Additional experiments are necessary to confirm the participation of ROS in the AQP4 dynamics. The specific inhibition of sources of ROS like NOX-2 or iNOS and the use of AQP4 KO animals in models of brain injury associated with edema will help to elucidate the molecular mechanism of the involvement of AQP4 under injury conditions. It is necessary to understand the regulation of AQP4 at the level of mRNA and protein expression, as well as the contribution of proteins related to AQP4 distribution in the cell membrane such as CaM or Cav-1 that are critical in the role of AQP4 during brain injury.

Despite the relevance of these data, there is still a lack of information on the mechanisms responsible for the neuroprotective action of the ECS. Particularly, the role of oxidative stress and the signaling pathways involved in the ECS action. Transgenic animals, specifically those with modifications for CB1 and CB2 receptors, offer invaluable models for unraveling the distinct roles these receptors play in ROS production, both in the healthy brain and after brain injury. These models provide a unique opportunity to investigate the endocannabinoid system influences on redox equilibrium. Interestingly, the activation of CB receptors has been linked to oxidative stress and AQP4 expression. There are few reports associating ECS with the expression and activity of AQP4 during brain injury. Understanding the molecular mechanisms by which the ECS regulates the expression and activity of AQP4 under conditions of acute damage could lead to novel approaches for the development of potential therapeutic tools directed to treat brain edema and neuronal damage.

## Data Availability

Not applicable.
